# Resistance of Bovine Spongiform Encephalopathy (BSE) Prions to Inactivation

**DOI:** 10.1371/journal.ppat.1000206

**Published:** 2008-11-14

**Authors:** Kurt Giles, David V. Glidden, Robyn Beckwith, Rose Seoanes, David Peretz, Stephen J. DeArmond, Stanley B. Prusiner

**Affiliations:** 1 Institute for Neurodegenerative Diseases, University of California San Francisco, San Francisco, California, United States of America; 2 Department of Neurology, University of California San Francisco, San Francisco, California, United States of America; 3 Department of Epidemiology and Biostatistics, University of California San Francisco, San Francisco, California, United States of America; 4 Department of Pathology, University of California San Francisco, San Francisco, California, United States of America; 5 Department of Biochemistry and Biophysics, University of California San Francisco, San Francisco, California, United States of America; University of Alberta, Canada

## Abstract

Distinct prion strains often exhibit different incubation periods and patterns of neuropathological lesions. Strain characteristics are generally retained upon intraspecies transmission, but may change on transmission to another species. We investigated the inactivation of two related prions strains: BSE prions from cattle and mouse-passaged BSE prions, termed 301V. Inactivation was manipulated by exposure to sodium dodecyl sulfate (SDS), variations in pH, and different temperatures. Infectivity was measured using transgenic mouse lines that are highly susceptible to either BSE or 301V prions. Bioassays demonstrated that BSE prions are up to 1,000-fold more resistant to inactivation than 301V prions while Western immunoblotting showed that short acidic SDS treatments reduced protease-resistant PrP^Sc^ from BSE prions and 301V prions at similar rates. Our findings argue that despite being derived from BSE prions, mouse 301V prions are not necessarily a reliable model for cattle BSE prions. Extending these comparisons to human sporadic Creutzfeldt-Jakob disease and hamster Sc237 prions, we found that BSE prions were 10- and 10^6^-fold more resistant to inactivation, respectively. Our studies contend that any prion inactivation procedures must be validated by bioassay against the prion strain for which they are intended to be used.

## Author Summary

“Mad cow” disease, formally known as bovine spongiform encephalopathy (BSE), belongs to a family of diseases affecting humans and a number of commercially important animal species. These diseases are not spread by bacteria or viruses, but by infectious proteins, termed “prions.” Prions are known to be very difficult to inactivate, but little is known about the relative difficulty of inactivation for prions from different species. Here, we studied the inactivation of BSE prions and compared it to the inactivation of prions from humans, mice, and hamsters. We used highly sensitive, genetically engineered mouse models to detect low levels of infectivity. We then quantified the levels of inactivation for a range of treatments and calculated differences between prions from different species. We found that naturally occurring BSE prions can be up to 1 million times more difficult to inactivate than the most commonly used hamster prions. BSE prions were also 1,000 times more difficult to inactivate than a mouse prion that was thought to be a surrogate for BSE prions. This study demonstrates that prion inactivation procedures need to be validated directly against the prion strains for which they are intended to be used.

## Introduction

Prions are proteinaceous infectious particles that cause invariably fatal neurodegenerative diseases, including Creutzfeldt-Jakob disease (CJD) and kuru in humans, bovine spongiform encephalopathy (BSE), chronic wasting disease (CWD) in deer and elk, and scrapie in sheep and goats. The only known component of the prion is an alternatively folded isoform, denoted PrP^Sc^, of the prion protein (PrP). The PrP^Sc^ isoform is derived from the normal, cellular PrP, denoted PrP^C^, through a poorly understood process. Accumulation of PrP^Sc^ in the central nervous system results in spongiform changes and death.

Distinct prions strains often exhibit different incubation periods and patterns of neuropathological lesions. Strain characteristics are generally retained upon intraspecies transmission, but may change on transmission to another species [1]–[4]. For many years, the biological properties of prion strains were thought to be encoded by a nucleic acid [5] but none was found. Data from numerous studies offer convincing evidence that strain-specified information is enciphered in the conformation of PrP^Sc^ [3], [6]–[11].

Among the characteristics that distinguish prion strains is resistance to inactivation. The extreme resistance of prions was first noted when numerous sheep immunized against looping-ill virus developed scrapie. The inoculum had been prepared from pooled sheep brains, spinal cords, and spleens, treated with formalin and certified free of viruses [12]. Later, the resistance of prions to both ionizing and ultraviolet radiation was found [13],[14]. As preparations of Sc237-infected Syrian hamster brain were progressively enriched for scrapie infectivity, procedures that modified proteins were found to inactivate the samples, while those that modified nucleic acids had no effect [15]. The results of numerous earlier studies concluded that protein denaturants were effective at reducing infectivity titers [16] but that complete inactivation required harsh conditions [17],[18].

The BSE epidemic and almost 200 cases of variant CJD in the United Kingdom as well as the growing problem of CWD in the United States have raised concerns about the spread of prion infectivity. These diseases highlight the need to decontaminate effectively meat processing equipment and other instrumentation of prions. Procedures used for routine sterilization of surgical instruments do not inactivate prions, which led to the development of stringent recommendations for reprocessing instruments used on known or suspected CJD cases [19]. These recommendations include autoclaving at 121°C in the presence of 1 M sodium hydroxide, or soaking in 2% sodium hypochlorite for 1 h. However, these highly corrosive procedures can cause significant damage to surgical instruments [20],[21] and therefore, are rarely used. Other less stringent procedures, such as autoclaving at 134°C for 18 min, are also included in the recommendations although it is acknowledged that these may not completely remove infectivity [19]. Moreover, the recommendations for prion inactivation are based on rodent-passaged prion isolates, because measuring infectivity in more relevant species is extremely expensive and time consuming. The development of transgenic (Tg) mice expressing a chimeric human/mouse PrP that are highly susceptible to human prions provided a sensitive model system [22]. Bioassay of human sporadic (s) CJD prions in these Tg mice demonstrated that sCJD prions are significantly more resistant to inactivation, by sodium dodecyl sulfate (SDS) at acidic pH, than hamster Sc237 prions [23].

We report here on the inactivation of BSE prions and a mouse-passaged BSE strain, termed 301V. The initial transmission of BSE prions to wild-type (*wt*) mice gave rise to multiple strains, one of which (301V) had a shorter incubation period in mice expressing the PrP-B polymorphic isoform [24],[25]. Even though 301V has been passaged in mice, it has been assumed by some authors to be a model for the original BSE strain [26]. Due to the major logistical problems of performing inoculation experiments on cattle, we chose highly sensitive Tg mouse models expressing either bovine (Bo) PrP or mouse (Mo) PrP-B. Inactivation of BSE and 301V prions was accomplished by varying the SDS concentration, the pH and temperature. Infectivity was measured using Tg mouse lines that are highly susceptible to either BSE or 301V prions. Bioassays demonstrated that BSE prions are up to 1,000-fold more resistant to inactivation than 301V prions while Western immunoblotting showed that short acidic SDS treatments reduced protease-resistant PrP^Sc^ from BSE prions and 301V prions at similar rates. We also found that BSE prions are 10- and 1,000,000-fold more resistant to inactivation than sCJD or hamster Sc237 prions, respectively. The hamster Sc237 strain is identical to the 263K strain used by other investigators [27],[28]. Our studies contend that prion inactivation procedures must be validated by bioassays against the prion strain for which they are intended to be used.

## Materials and Methods

### Inocula

Mouse 301V, originally a gift from Dr. H. Fraser, was serially passaged in B6.I mice expressing the mouse PrP-B polymorphic isoform [29]. Cattle BSE was from a histopathologically confirmed Hereford bull, case PG31/90, reported previously [30]. This BSE sample showed an electrophoretic banding pattern similar to other BSE samples [31], arguing that it is a “classical” BSE strain. Human sCJD and hamster Sc237 samples were previously described [23].

Brain homogenates (10% w/v) were prepared in Ca^2+^ and Mg^2+^-free phosphate buffered saline (PBS; Invitrogen, Carlsbad, CA), by either repeated extrusion through successively smaller needles, via tissue disruption using stainless steel beads in a Mini-BeadBeater-8 apparatus (BioSPec, Bartlesville, OK), or with a PowerGen 125 homogenizer, as previously described [23],[32],[33].

Four-millimeter segments of stainless steel suture wire (Ethicon, Cornelia, GA) were incubated in brain homogenate as previously described [23].

### Prion inactivation treatments

Acidic SDS treatments were performed by incubating 50-µl aliquots of brain homogenate with 2× concentration of acidic SDS, for various durations (30 seconds to 18 h) and temperatures (65, 121, or 134°C). Samples were then neutralized to stop the inactivation, and either PrP^Sc^ levels were detected by Western blotting or samples were prepared for infectivity bioassay.

### PrP^Sc^ detection by immunoblotting

Treated samples were neutralized and PrP^Sc^ was determined by limited proteinase K (PK) digestion and immunoblotting as described previously [34]. After incubations, 890 µl of cold neutralization buffer [4% Sarkosyl; 100 mM 4-(2-hydroxyethyl)piperazine-1-ethanesulfonic acid (HEPES; pH 7.5); 200 mM NaCl] was added to each sample. Protease digestion was performed with 20 µg of PK/ml (Invitrogen, Carlsbad, CA) for 1 h at 37°C. Digestions were terminated by the addition of 10 µl of 0.1 M phenylmethylsulfonyl fluoride in absolute ethanol (Roche, Indianapolis, IN). Digested samples were then resuspended in a final volume of 200 µl using neutralization buffer mixed with 4× LDS sample buffer (Invitrogen) and 10× reducing agent (Invitrogen). Samples were boiled for 10 min at 100°C prior to electrophoresis on NuPAGE Bis-Tris 4–12% gels (Invitrogen). Proteins were transferred onto a PVDF membrane using the iBlot dry blotting system (Invitrogen).

Material was eluted from wires by incubating in 10% SDS, 20 mM dithiothreitol (DTT) at 100°C for 5 min. The resulting solution was loaded onto a Minifold slot blot apparatus (Schleicher & Schuell, Florham Park, NJ) containing a PVDF membrane presoaked in ethanol.

Membranes were blocked for 30 min with 5% nonfat milk in TBST (100 mM Tris-HCl, pH 8.0; 150 mM NaCl; 0.5% Tween-20) and incubated with HRP-conjugated HuM-P monoclonal antibody diluted to 0.05 µg/ml in TBST for 1 h at room temperature. Following three 10-min washes in TBST with water rinses, membranes were developed using an enhanced chemiluminescent (ECL) detection system (Amersham, Piscataway, NJ), followed by exposure to X-ray film. Densitometry was performed with ImageJ Software (NIH, Bethesda, MD).

### Bioassay in transgenic mice

To directly measure infectivity of BSE prions, we performed bioassays in homozygous Tg mice expressing bovine PrP, Tg(BoPrP^+/+^)4092/*Prnp* ^0/0^, described previously [35]. In order to develop a sensitive and rapid model for the detection of 301V prions, transgenic mice expressing MoPrP-B at ∼4-fold that of *wt* mice were generated. The open reading frame of MoPrP-B was cloned into the Cos.tet vector, then microinjected into *Prnp* ^0/0^ zygotes, as previously described [36]. Potential founder mice were identified by dot blot of genomic DNA and binding of a radiolabeled probe complementary to the 3′ untranslated region of the vector [36]. From a founder with a high copy number, we developed a hemizygous line, denoted Tg(MoPrP-B)2091/*Prnp* ^0/0^ mice.

All mice were inoculated intracerebrally with a 30-µl aliquot of sample. Samples treated with acidic SDS were neutralized by addition of an equal volume of neutralization buffer, then diluted 10-fold in 5% BSA in PBS, to minimize nonspecific binding to the vial. For serial dilution of brain homogenate, three independent replicates of 10 ten-fold serial dilutions of 301V prions were prepared in *Prnp* ^0/0^ brain homogenate, and each inoculated into groups of four mice. Stainless-steel wires coated in brain homogenate were implanted as previously described [23]. Mice were monitored for onset of clinical signs of prion disease, as described [29],[32].

### Statistical analysis

While incubation periods are commonly reported as the mean time from inoculation to onset of disease, analysis of this duration for samples harboring low infectivity titers requires more rigorous analysis [23],[37]. For the studies reported here, a range of survival analysis techniques was employed to enable censored data to be included in the calculations. Median incubation periods were calculated using the Kaplan-Meier function [38], and 95% confidence intervals (ci) were determined [39]. When an upper 95% ci could not be calculated by survival analysis techniques, it was estimated by binomial interpolation, using the times to disease onset of sick animals. To determine how hazard (disease onset) varied with time, we developed both semi-parametric Cox [40] and fully parametric Weibull [41] regression models from survival data for serially diluted brain homogenate. We also studied how hazard varied by dilution, as a subjective check of the model, using a seven-parameter spline. To determine log reduction, the survival data for positive control and treated samples were compared to the regression models. When comparing the effect of a given treatment for two strains, individual Cox regression models were stratified by strain [42]. Such survival analysis tools have the advantage of calculating a measure of variability (expressed as 95% ci) for the comparisons.

Specifically, to determine log reduction, we used terms for log dilution and treatment: λ_jk_(t) = λ_k_(t) exp(β_k_ log dilution+δ_jk_ treatment), where the subscripts k and j denote strain and treatment, respectively. The equivalent log reduction *d*_jk_ = −δ_jk_/β_k_ for the j treatment was then determined separately for each strain. The equivalent log reduction for a treatment is then the dilution for which the Cox model predicts the hazard of illness equals the hazard in the treated animals. We determined 95% ci for d_j0_, d_j1_, and their difference using profile likelihood ratio-based confidence intervals. All calculations were performed with Stata 9 (Stata Corp., College Station, TX) and R (R Foundation for Statistical Computing, Vienna, Austria).

## Results

### Prion strains have unique electrophoretic characteristics

**Figure 1:**
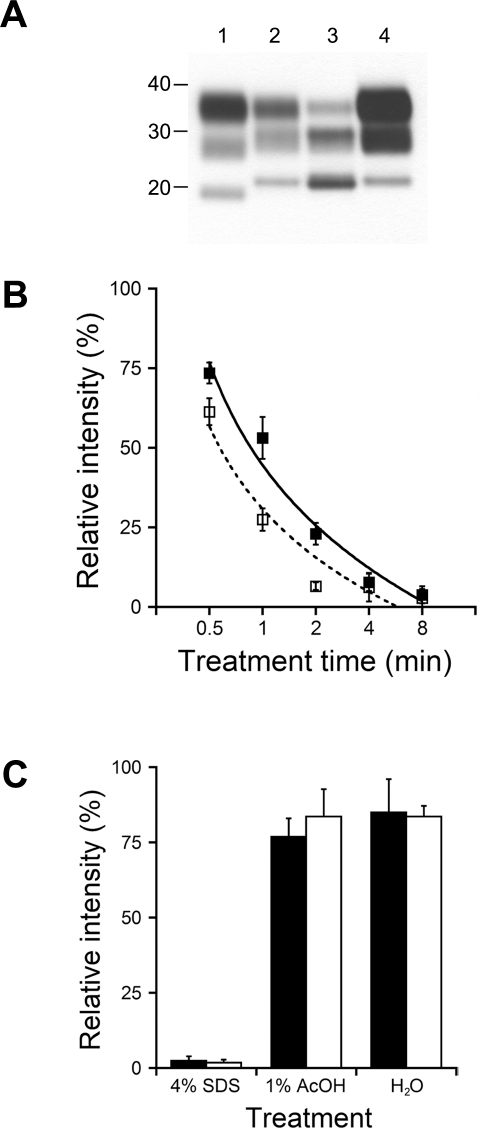
Strain characterization and PrP^Sc^ levels before and after acidic SDS treatment, by gel electrophoresis. (A) Western blot of samples after limited digestion with proteinase K. Brain homogenates (0.5 mg equivalent loaded per lane) were prepared from a cow with BSE (lane 1), mouse infected with 301V (lane 2), human with sCJD (lane 3), and hamster infected with Sc237 (lane 4) prions. (B) Residual PrP^Sc^ levels of mouse 301V (filled symbols) and cattle BSE (open symbols) after treatment with 4% SDS–1% AcOH at 65°C, for 0.5 min, 1 min, 2 min, 4 min, or 8 min, as indicated. (C) Residual PrP^Sc^ levels of mouse 301V (filled bars) and cattle BSE (open bars) after treatment with 4% SDS, 1% AcOH, or water for 18 h at 65°C, as indicated. For (B) and (C), residual PrP^Sc^ levels were quantified by densitometry and compared to untreated controls. Values represent means with error bars representing standard deviations, from at least three independent measurements.

Western immunoblotting of the four prion strains in this study revealed distinct electrophoretic profiles. While unglycosylated PrP^Sc^ of BSE migrated to 19 kDa (Figure 1A, lane 1), those of 301V, sCJD, and Sc237 had a molecular mass of 21 kDa (Figure 1A, lanes 2–4). While the host species may influence the glycoforms ratios, it is interesting to note that mouse 301V and hamster Sc237 gave similar banding patterns for un-, mono-, and diglycosylated PrP^Sc^.

### PrP^Sc^ of mouse 301V and cattle BSE show similar resistance to acidic SDS

We tested the resistances of PrP^Sc^ of cattle BSE and mouse 301V to acidic SDS. Treatment with 4% SDS–1% AcOH for 0.5 to 8 min at 65°C demonstrated a clear time dependence for reduction of PrP^Sc^ levels for both strains. Although the rates of PrP^Sc^ reduction were similar, mouse 301V PrP^Sc^ was marginally more resistant to acidic SDS than cattle BSE PrP^Sc^ (Figure 1B). Longer treatment durations with acidic SDS reduced the PrP^Sc^ signals below detectable levels for both strains, as did treatment with 4% SDS alone for 18 h (Figure 1C). In comparison, incubation with either water or 1% AcOH for 18 h at 65°C had little effect on PrP^Sc^ levels from either strain (Figure 1C).

### Inactivation of prions measured by bioassay

To determine more accurately the differences in inactivation of prion strains, we turned to bioassays, which are the only direct method of determining infectivity and have a much larger dynamic range than Western blots. We previously found that a two-step treatment protocol, of 4% SDS at 65°C followed by SDS at acidic pH, was the most effective at inactivating prions [23]. Cattle BSE and mouse 301V prions were treated with SDS at acidic pH at 65°C and 134°C for a range of time periods. Additional samples were incubated at 65°C in either water, 1% acetic acid, or 4% SDS, for 18 h; or autoclaved at 134°C in the absence of acidic SDS. All experiments were performed in triplicate and inoculated into independent groups of eight mice (Table 1).

**Table 1:** Inactivation of prions in brain homogenates.

Treatment	Mouse 301V in Tg2091 mice		Cattle BSE in Tg4092 mice	
	IP (95% ci)	Sick (%)	IP (95% ci)	Sick (%)
Negative control	>600	0	>500	0
Positive control	88 (81, 97)	100	272 (256, 277)	100
ddH_2_O, 18 h, 65°C	95 (95, 99)	100	298 (286, 312)	100
1% AcOH, 18 h, 65°C	102 (99, 106)	100	316 (298, 368)	100
4% SDS, 18 h, 65°C	200 (151, >600)	67	371 (347, 403)	91
4% SDS–1% AcOH, 30 min, 65°C	305 (244, >600)	57	>500	18
4% SDS–1% AcOH, 2 h, 65°C	>600	22	>500	5
4% SDS–1% AcOH, 18 h, 65°C	>600	13	>500	0
Untreated, 15 min, 134°C	>600	14	>500	18
Untreated, 30 min, 134°C	>600	0	>500	10
Untreated, 2 h, 134°C	>600	0	>500	0
4% SDS–1% AcOH, 15 min, 134°C	>600	0	>500	0
4% SDS–1% AcOH, 30 min, 134°C	>600	5	>500	0
4% SDS–1% AcOH, 2 h, 134°C	>600	0	>500	0

Median incubation period (IP) in days, 95% confidence intervals (ci), and percentage of animals succumbing to prion disease (24 mice per treatment) were calculated by using Kaplan-Meier analysis.

Mouse 301V prions were only partially inactivated by acidic SDS at 65°C for 30 min: the median incubation period was significantly extended from 88 d (95% ci: 81, 97) to 305 d, but the majority of the animals still succumbed to disease. Treatment at 65°C for 18 h further decreased infectivity titers, evidenced by fewer animals (13%) becoming ill, but failed to inactivate mouse 301V prions beyond the level of detectability. A similar incidence of sick animals (14%) was seen after autoclaving at a temperature of 134°C for 15 min in the absence of acidic SDS, but an equivalent treatment with acidic SDS led to complete survival. Interestingly, treatment with SDS alone at 65°C for 18 h led to a significant reduction in mouse 301V infectivity.

Similar to 301V, cattle BSE prions were also resistant to inactivation. Treatment with acidic SDS at 65°C for 30 min produced a significant reduction in infectivity, with over 80% of the mice remaining healthy for the duration of the experiment. Increasing the treatment time increased the level of inactivation: after 18 h at 65°C with acidic SDS, no mice succumbed to disease. Autoclaving at 134°C alone (in the absence of acidic SDS) reduced BSE infectivity, but complete inactivation required 2 h. In the presence of acidic SDS, complete inactivation was achieved in 15 min at 134°C, as observed with mouse 301V.

### Developing a standard by serial dilution

**Figure 2:**
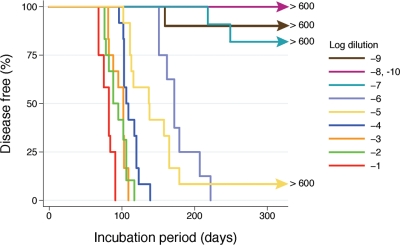
Kaplan-Meier survival curves for serially diluted 301V prions bioassayed in Tg(MoPrP-B)2091/*Prnp* ^0/0^ mice. Three independent 10% brain homogenates were serially diluted 10-fold and each inoculated into 4 mice, which were then monitored for clinical symptoms of prion disease. No significant differences were observed among the three dilution series and the data were therefore combined. Incubation period is the number of days from inoculation to manifestation of clinical symptoms. Mice with intercurrent illnesses were censored at the time of euthanasia.

Due to the different levels of sensitivity of *wt* and Tg mice to different prion strains, direct comparison of survival is not an accurate method. Rather, the log reduction in infectivity for each strain must be calculated by comparison to serially diluted samples of the same strain. Tg2091 mice were inoculated with three sets of independent dilutions of mouse 301V prions; no statistically significant difference was found between the sets, so the data were combined for further analyses. As the samples of 301V prions were titrated from 10^−1^ to 10^−6^ dilution of 10% brain homogenate, the median incubation periods increased from 82 d (95% ci: 69, 90) to 172 d (95% ci: 151, 207). At the 10^−7^ dilution, only a portion (<25%) of the animals succumbed to disease. For both the 10^−8^ and 10^−10^ dilutions, no animals became ill. However, for the 10^−9^ dilution, one mouse showed clinical signs at 159 d after inoculation, and prion disease was confirmed by Western immunoblotting (Figure 2).

The incubation periods of serially diluted cattle BSE, human sCJD and hamster Sc237 prions [23],[35] were also analyzed using survival-analysis techniques (Figure S1).

### Fitting a mathematical model of the serial dilution data

**Figure 3:**
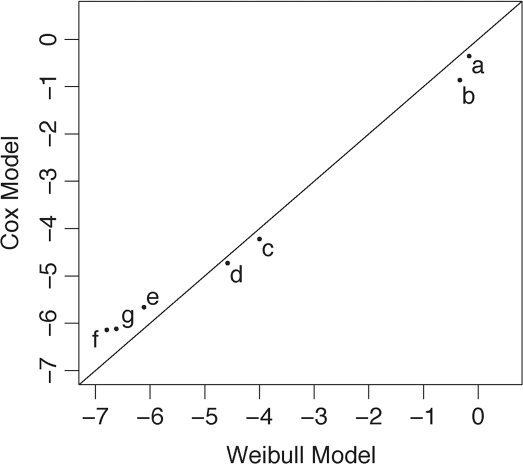
Comparison of mathematical models derived from serial dilution data. Calculations of log reduction upon inactivation treatment, using the Weibull (abscissa) and Cox (ordinate) models, produce similar results. Serial dilutions of 301V-infected brain homogenate were modeled using the Cox and Weibull regression methods and log reduction was calculated for each treatment by both methods. Samples were treated as follows: ddH_2_O, 65°C, 18 h (a); 1% AcOH, 65°C, 18 h (b); 4% SDS, 65°C, 18 h (c); 4% SDS–1% AcOH at 65°C for 30 min (d), 2 h (e), and 18 h (f); autoclaved at 134°C for 15 min (g). The correlation coefficient to the line of identity is 0.99.

Two statistical methods were used to model the serially diluted mouse 301V prions: (i) a semi-parametric Cox model, which considers relative rate of disease onset, and (ii) the fully parametric Weibull model, which makes more detailed parametric assumptions of how rate of disease varies over time. For each inactivation treatment, log reductions were calculated by both methods. A plot of the log reductions determined using the Weibull model against those calculated by the Cox model demonstrates that the two methods produce very similar results (Figure 3), with a correlation coefficient of 0.99 to the line of identity. We therefore concluded that the additional assumptions of the Weibull model did not lead to greater accuracy, and used the Cox model for subsequent calculations. To test the assumption that the rate of developing disease was linear over time, we examined how hazard rate varied across dilutions, using penalized regression splines [43]. The multi-parameter spline interpolation fitted very closely a two-parameter linear relationship; thus, we found no evidence of non-linearity in the observable range, validating our choice of model (Figure S2).

### Quantification of prion inactivation

Values of log reduction in infectivity were calculated for each treatment and strain by comparing survival after treatment with a positive control, using the appropriate Cox model (Table 2). The limit of detectability for each set of experiments is a feature of the infectivity of the positive control and the sensitivity of the transgenic line. In the inactivation experiments based on 4% SDS, the maximum log reduction detectable for each of the four strains was 8.1, 4.0, 7.2, and 7.2 for mouse 301V, cattle BSE, human sCJD, and hamster Sc237, respectively. When all mice survive following inoculation with a sample subjected to an inactivation treatment, the application of survival-analysis techniques enabled the calculation of a lower confidence interval for the level of inactivation.

**Table 2:** Quantification of log reduction in infectivity, with 95% confidence intervals for various prion strains.

Treatment	301V prions	BSE prions	sCJD prions	Sc237 prions
ddH_2_O, 18 h, 65°C	0.5 (0.0, 1.0)	0.9 (0.5, 1.2)	2.4 (1.9, 2.9)	0.5 (0.0, 1.1)
1% AcOH, 18 h, 65°C	1.0 (0.5, 1.5)	1.4 (1.0, 1.7)	1.5 (1.2, 1.9)	0.8 (0.4, 1.3)
4% SDS, 18 h, 65°C	4.3 (3.8, 5.0)	1.7 (1.4, 2.1)	3.3 (2.7, 4.0)	2.5 (2.0, 3.1)
4% SDS–1% AcOH, 30 min, 65°C	4.8 (4.2, 5.5)	2.9 (2.4, 3.7)	4.9 (3.9, 6.7)	>7.2 (6.4, >7.2)
4% SDS–1% AcOH, 2 h, 65°C	5.8 (5.0, 6.8)	3.6 (2.7, 5.3)	4.2 (3.5, 5.2)	>7.2 (6.4, >7.2)
4% SDS–1% AcOH, 18 h, 65°C	6.3 (5.2, 7.8)	>4.0 (3.1, >4.0)	>7.2 (4.3, >7.2)	>7.2 (6.4, >7.2)
Untreated, 15 min, 134°C	6.2 (5.2, 7.8)	2.9 (2.3, 3.7)	>7.2 (4.3, >7.2)	7.1 (5.6, >7.2)
Untreated, 30 min, 134°C	>8.1 (6.5, >8.1)	3.2 (2.5, 4.3)	>7.2 (4.3, >7.2)	>7.2 (6.4, >7.2)
Untreated, 2 h, 134°C	>8.1 (6.6, >8.1)	>4.0 (3.1, >4.0)	>7.2 (4.3, >7.2)	>7.2 (6.4, >7.2)
4% SDS–1% AcOH, 15 min, 134°C	>8.1 (6.8, >8.1)	>4.0 (3.1, >4.0)	>7.2 (4.3, >7.2)	>7.2 (6.4, >7.2)
4% SDS–1% AcOH, 30 min, 134°C	7.4 (5.8, >8.1)	>4.0 (3.1, >4.0)	>7.2 (4.4, >7.2)	>7.2 (6.4, >7.2)
4% SDS–1% AcOH, 2 h, 134°C	>8.1 (6.5, >8.1)	>4.0 (3.1, >4.0)	>7.2 (4.4, >7.2)	>7.2 (6.4, >7.2)

### Comparing inactivation between strains

The log difference between strains for a given treatment is simply the arithmetic difference between log reductions, for a given treatment, for the two strains. However, to determine levels of significance and confidence intervals for these differences, the comparisons were stratified using individual Cox models for each strain. For treatments that led to low levels of prion inactivation (H_2_O or AcOH, 18 h, 65°C), mouse 301V prions and cattle BSE prions had only slightly different inactivation profiles, with a log difference of −0.4 (95% ci: −1.0, 0.2; *p* = 0.1). At higher levels of inactivation, mouse 301V prions were significantly less resistant to inactivation than cattle BSE prions. Treatment with acidic SDS at 65°C for 30 min or 2 h, gave log differences of 1.9 (95% ci: 1.0, 2.8; *p*<0.001) and 2.2 (95% ci: 0.3, 3.6; *p* = 0.03) respectively; autoclaving at 134°C for 15 min in the absence of acidic SDS gave a log difference of 3.3 (95% ci: 2.0, 4.9; *p*<0.001). For all other inactivation treatments one or both the strains had complete survival of mice and therefore log reduction for the treatments cannot be calculated. At higher levels of inactivation, mouse 301V prions are 100 to 1000-fold less resistant to inactivation than cattle BSE prions.

Similar treatments on sCJD prions showed them to be approximately 10-fold easier to inactivate than BSE prions, however many treatments led to inactivation beyond the limit of detectability for sCJD prions. With Sc237 prions the 4% SDS treatments were even more effective at resulting in complete survival of bioassay mice, and thus an inability to quantify differences between the strains. We therefore repeated the less effective 2% SDS–1% AcOH procedures on BSE prions and compared them with equivalent inactivation treatments on Sc237 and sCJD prions [23] (Table S1). For human sCJD, values for the log difference to cattle BSE range from non-significant to a log difference of 2.0 (95% ci: 0.7, 3.9; *p*<0.001) for acidic 4% SDS at 65°C for 30 min. In general there was about a 1 log difference, implying that human sCJD prions are ∼10-fold less resistant to inactivation that cattle BSE prions. For Sc237 prions, highly significant differences against cattle BSE prions were found for inactivation treatments with acidic 2% SDS at 65°C for 30 min and autoclaving at 121°C for 15 min in the absence of acidic SDS, giving log differences of 4.0 (*p*<0.001) and 5.1 (*p*<0.001) respectively. In both cases the upper 95% ci cannot be calculated due to the limit of the level of detectability. In nearly all other treatments hamster Sc237 prions were inactivated beyond the level of detectability, i.e. greater than 8.4 log reduction, even when BSE infectivity was only reduced by approximately 2 logs, suggesting a difference of approximately 6 logs. Hamster Sc237 prions were at least 10,000-fold and in some cases up to 1,000,000-fold, less resistant to inactivation than cattle BSE prions.

### Inactivation of prions bound to stainless steel

Stainless-steel wires soaked in brain homogenates containing either cattle BSE or mouse 301V prions were subjected to a similar series of inactivation procedures as the brain homogenates. Wires were implanted into the brains of the respective Tg mouse lines, which were then monitored for onset of prion disease (Table 3). To determine the amount of PrP bound to the wires, 20 additional prion-soaked wires were prepared in the same manner, including the repeated washing steps, then boiled with 10% SDS and 20 mM DTT to elute the bound material. PrP was detected by slot blot and compared to a serially diluted, prion-infected brain homogenate. We determined that the amount of PrP bound to each wire was equivalent to that in ∼250 ng of brain tissue. It is not possible to confirm that all protein was eluted from the wires, so this must be taken as a minimum value.

**Table 3:** Inactivation of prions bound to stainless steel.

Treatment	Mouse 301V in Tg2091 mice		Cattle BSE in Tg4092 mice	
	IP (95% ci)	Sick (%)	IP (95% ci)	Sick (%)
Negative control	>600	0	>500	0
Positive control	113 (110, 116)	100	350 (336, 357)	100
ddH_2_O, 18 h, 65°C	116 (109, 124)	100	357 (349, 368)	100
1% AcOH, 18 h, 65°C	117 (111, 125)	100	354 (336, 368)	91
4% SDS, 18 h, 65°C	127 (123, 151)	100	368 (354, 398)	100
4% SDS–1% AcOH, 30 min, 65°C	267 (183, 491)	73	>500	42
4% SDS–1% AcOH, 2 h, 65°C	>600	33	>500	26
4% SDS–1% AcOH, 18 h, 65°C	410 (215, >600)	58	>500	4
Untreated, 15 min, 134°C	161 (151, 200)	96	384 (343, 456)	84
Untreated, 30 min, 134°C	438 (232, >600)	57	375 (361, 386)	100
Untreated, 2 h, 134°C	>600	14	420 (398, 452)	89
4% SDS–1% AcOH, 15 min, 134°C	>600	5	>500	0
4% SDS–1% AcOH, 30 min, 134°C	>600	0	>500	0
4% SDS–1% AcOH, 2 h, 134°C	>600	0	>500	0

Median incubation period (IP) in days, 95% confidence intervals (ci), and percentage of animals succumbing to prion disease (24 mice per treatment) were calculated by using Kaplan-Meier analysis.

Inactivation treatments on prion-coated wires produced only moderate reductions in the level of infectivity. For example, autoclaving 301V-coated wires at 134°C for 15 min extended the median incubation period from 113 d to 161 d, and 96% of animals developed disease (Table 3). In comparison, the same procedure on 301V-infected brain homogenate extended the median incubation period from 88 d to over 600 d, and only 14% of mice succumbed to disease (Table 1). To remove all detectable infectivity from wires, acidic SDS treatment at elevated temperatures was required. Similar results were found for cattle BSE prions (Table 3, Table S2).

## Discussion

The application of survival analysis techniques to prion incubation periods provides a more accurate representation of the data. The distribution of animals succumbing to disease is rarely symmetrical, and not all animals become sick at low levels of infectivity. These factors are reflected by reporting median incubation periods and asymmetric 95% ci, which are by definition wider than standard deviation or standard error of the mean. These techniques also allow us to quantify inactivation using different Tg model systems, in an unbiased way.

We previously demonstrated that human CJD prions were significantly more resistant to inactivation than hamster Sc237 prions [23]. This differential inactivation of prion strains has also been observed by others. The phenolic detergent Environ LpH was shown to inactivate all detectable infectivity of hamster Sc237 prions [44],[45], but only modestly reduced infectivity of mouse RML prions [46].

Rodent-passaged prion strains are widely used in prion research. While these have been invaluable for understanding prion biology, great care must be taken in extrapolating any characteristics of these prions back to the original species and strain from which they were derived. Recommendations for the inactivation of human CJD prions were partly based on experiments on adapted strains of CJD passaged in mice [47], hamsters [48], and guinea pigs [49]. However, since the sequence of PrP^Sc^ is encoded by the host in which the prions were last passaged, these “CJD” strains are actually mouse, hamster and guinea pig prions, respectively, which may exhibit different resistances to inactivation compared to the human CJD prions from which they were originally derived.

Gel electrophoresis can be used to identify the PK-resistant core of PrP^Sc^, which can differ between strains. This technique was used to classify human prion strains based on the size of the unglycosylated, protease-resistant PrP^Sc^ fragments: type 1 results from cleavage at residue 82 and has a fragment size of 21 kDa and type 2 results from cleavage at residue 97 and has a fragment size of 19 kDa [50]. Despite the assumption that 301V is representative of the BSE strain from which it was derived, we observed a difference in the sizes of the unglycosylated bands between the two strains (Figure 1), which is consistent with previously published results. A mixture of N-terminal sequences was obtained from the 301V strain, starting with residue 81 [51]. In contrast, BSE was not recognized by an antibody directed against residues 89–104, suggesting the major cleavage point is within this region [52]. Furthermore, cattle BSE and mouse 301V PrP^Sc^ have different susceptibilities to acidic SDS (Figure 1B, Tables 1 and 2), suggesting that they are different strains.

We observed differences in the relative effectiveness of treatments on the two strains, as measured by Western blots and infectivity bioassay. In all cases, increasing the time and temperature of acidic SDS treatments led to reduced PrP^Sc^ levels and increased inactivation. For acidic SDS treatments of <8 min, Western blots of treated mouse 301V and cattle BSE prions showed similar levels of residual PrP^Sc^ (Figure 1B). In contrast, longer acidic SDS treatments demonstrated that cattle BSE prions were significantly more resistant to inactivation, up to 1,000-fold compared to mouse 301V prions. PrP^Sc^ levels of both 301V and BSE were relatively unchanged by exposure to water or 1% AcOH for 18 h at 65°C, compared to ∼10-fold reduction in infectivity as detected by bioassay. For both 301V and BSE prions, incubation with 4% SDS alone for 18 h at 65°C, or with 4% SDS–1% AcOH for 8 min at 65°C, reduced PrP^Sc^ to levels approaching the background signal in Western immunoblots, which implies a reduction of ≥2 logs (Figure 1C).

Western blotting is a rapid and convenient way of determining the level of PrP^Sc^, but it is limited by a dynamic range of ∼100-fold. It has long been known that the PrP^Sc^ level correlates with infectivity [53], but the precise relationship between the two remains to be determined. While PrP^Sc^ was originally defined as resistance to PK, we have since shown that there are both protease-resistant (r) and protease-sensitive (s) forms of PrP^Sc^ [54]. Western blotting only detects rPrP^Sc^, while the conformation-dependent immunoassay [8] and the amyloid seeding assay [55] are able to detect both rPrP^Sc^ and sPrP^Sc^.

The apparent discrepancies we observed between PrP^Sc^ level and infectivity for 301V and BSE prions may be due to several factors. First, the sPrP^Sc^ fraction, and its associated infectivity, may be variable between 301V and BSE. If 301V and BSE have differing amounts of rPrP^Sc^ and sPrP^Sc^, and at least some infectivity is associated with sPrP^Sc^, then Western blot results would not necessarily correlate with infectivity. Second, structural differences between the strains may result in different levels of inactivation. Presumably, the quaternary structure would be broken down first when inactivating prions, whereas the tertiary structure would have to be unfolded to diminish higher levels of infectivity. In such a case, mouse 301V prions may have an equally stable quaternary structure but less stable tertiary structure compared to cattle BSE prions.

Cattle or human prions passaged in rodents give rise to rodent prions. Mouse 301V prions are very resistant to inactivation [56]. Additionally, a human prion strain causing Gerstmann-Sträussler-Scheinker disease was passaged in mice; the resulting strain, termed M1000, was reported to be highly resistant to inactivation [57]. However, for both 301V and M1000, the relative susceptibilities to inactivation were not directly compared to their respective parent strains. We demonstrate here that mouse 301V and its parent strain, BSE, have different resistances to inactivation. Therefore, any extrapolation from a rodent-passaged prion strain to the original parent strain must be interpreted cautiously. A more suitable model for BSE prions may be BSE passaged in Tg(BoPrP) mice, which produce PrP^Sc^ with the BoPrP sequence as well as with the same electrophoretic mobility and glycosylation pattern as cattle BSE prions [58]. Infected brains from this line were recently shown to be suitable for evaluation of BSE tests [59]. However, these bovine prions are in the milieu of mouse brain homogenate rather than cattle brain homogenate, and it remains to be determined whether this has an effect on the inactivation characteristics.

As with the results reported previously for hamster Sc237 and human sCJD prions [23], the inactivation of cattle BSE and mouse 301V prions from stainless-steel surfaces is more difficult than inactivating similar levels of infectivity in brain homogenates. Stainless-steel wire as a model for surface contamination [60] provides a useful tool to study prion inactivation from surgical instruments or machinery used in slaughterhouses. After 4-mm wires were incubated in 10% brain homogenate, then washed extensively, we were able to elute PrP equivalent to ∼250 ng of brain tissue from each wire. Other investigators were able to elute substantially higher PrP levels from wires soaked in brain homogenate [61], but their wires were not exhaustively washed, as ours were. Previous studies, using very similar washing strategies, reported the inability to detect eluted PrP or other proteins from stainless-steel wires [62]. However, 2 M NaOH used in those studies may have hydrolyzed many of the proteins, including PrP.

In conclusion, we have quantified the relative resistance to inactivation of four prion strains from different species. Cattle BSE prions appear to be the most resistant strain studied. In comparison, human sCJD prions are approximately 10-fold less resistant to inactivation by SDS at neutral or acidic pH, or by heat alone. Mouse 301V prions are 100- to 1,000-fold less resistant, and hamster Sc237 prions are up to 1,000,000-fold less resistant to inactivation. As shown by our findings, prion inactivation based on rodent-passaged prion strains may not be effective against the naturally occurring strains for which they were developed.

## Supporting Information

Figure S1 Kaplan-Meier survival curves of 10-fold serial dilutions of (A) cattle BSE prions bioassayed in Tg(BoPrP^+/+^)4092/*Prnp* ^0/0^ mice; (B) human sCJD prions bioassayed in Tg(MHu2M,M165V,E167Q^+/+^)22372/*Prnp* ^0/0^ mice; (C) hamster Sc237 prions bioassayed in Tg(SHaPrP^+/+^)7/*Prnp* ^0/0^ mice. Three independent serial dilution experiments were performed for each strain; no significant differences were found between the replicates, so the data were combined.
(0.38 MB TIF)

Figure S2 Variation of hazard rate across serial dilutions for the 301V prion strain. The multi-parameter spline regression (black) is closely approximated by a linear relationship (gray).
(0.07 MB TIF)

Table S1 Inactivation of prion strains in brain homogenates.
(0.06 MB DOC)

Table S2 Inactivation of cattle BSE prions bound to stainless steel wires.
(0.04 MB DOC)
