# Supplementary figures and images for: Resistance of Bovine Spongiform Encephalopathy (BSE) Prions to Inactivation
Source: PLoS Pathog. 2008 Nov 14;4(11):e1000206. doi: 10.1371/journal.ppat.1000206 (PMC2576443; doi:10.1371/journal.ppat.1000206)

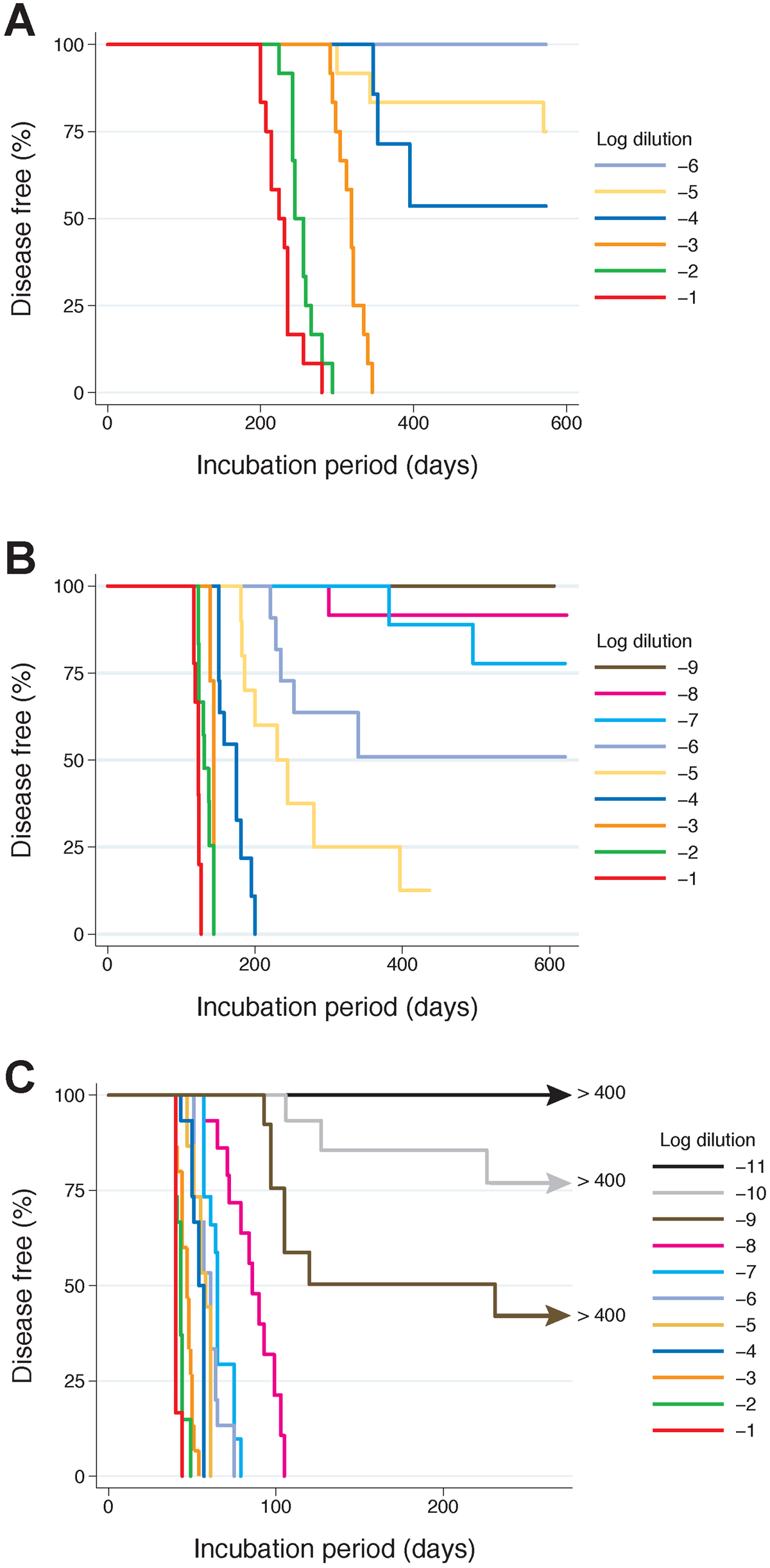

Supplement: Figure S1 — Kaplan-Meier survival curves of 10-fold serial dilutions of (A) cattle BSE prions bioassayed in Tg(BoPrP+/+)4092/Prnp 0/0 mice; (B) human sCJD prions bioassayed in Tg(MHu2M,M165V,E167Q+/+)22372/Prnp 0/0 mice; (C) hamster Sc237 prions bioassayed in Tg(SHaPrP+/+)7/Prnp 0/0 mice. Three independent serial dilution experiments were performed for each strain; no significant differences were found between the replicates, so the data were combined. (0.38 MB TIF) [file ppat.1000206.s001.tif]

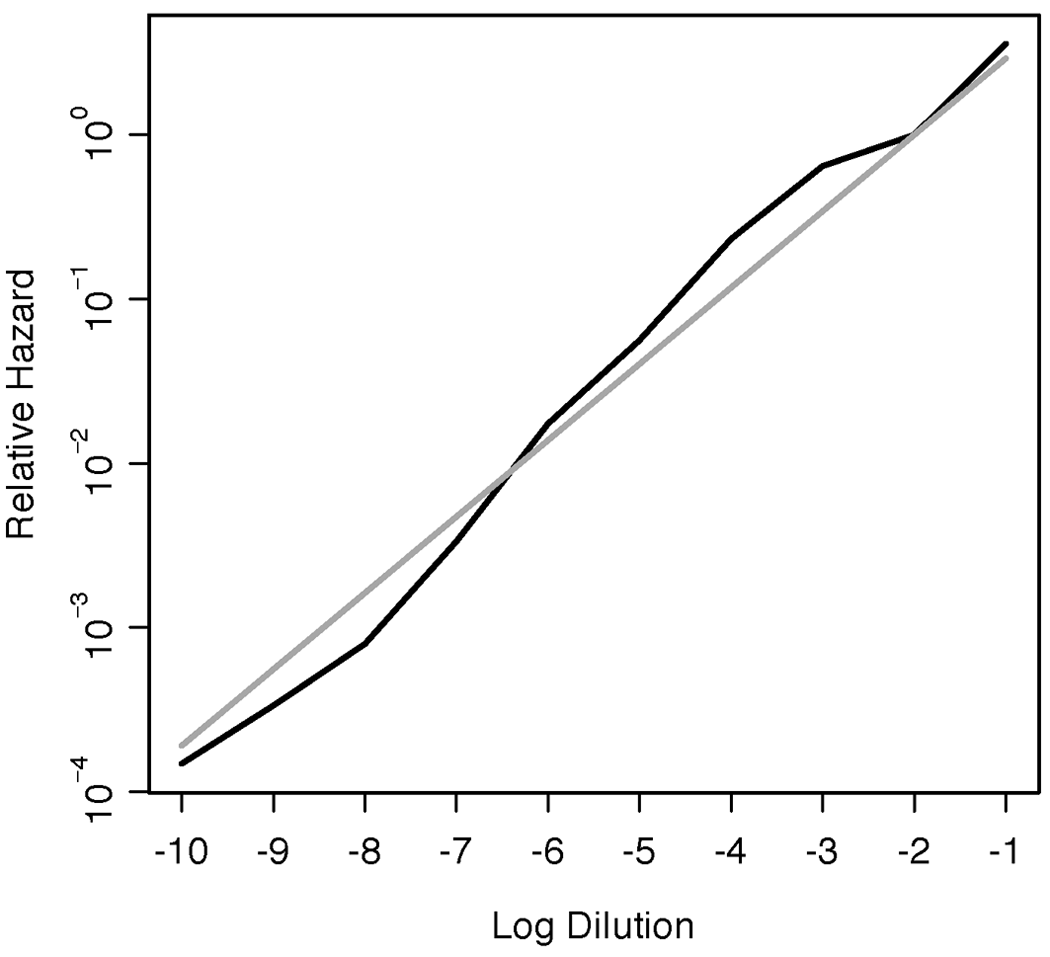

Supplement: Figure S2 — Variation of hazard rate across serial dilutions for the 301V prion strain. The multi-parameter spline regression (black) is closely approximated by a linear relationship (gray). (0.07 MB TIF) [file ppat.1000206.s002.tif]
