# Supplementary material for: Resistance of Bovine Spongiform Encephalopathy (BSE) Prions to Inactivation
Source: PLoS Pathog. 2008 Nov 14;4(11):e1000206. doi: 10.1371/journal.ppat.1000206 (PMC2576443; doi:10.1371/journal.ppat.1000206)
Supplement: Table S1 — Inactivation of prion strains in brain homogenates. (0.06 MB DOC) [file ppat.1000206.s003.doc]

Suppl. Table 1. Inactivation of prion strains in brain homogenates^a^

|  | BSE prions | | | sCJD prions*^b^* | Sc237 prions*^b^* |
| --- | --- | --- | --- | --- | --- |
| Treatment | IP (95% ci) | Sick (%) | Log reduction (95% ci) | Log reduction  (95% ci) | Log reduction  (95% ci) |
| **Negative control** | **> 500** | **0** |  |  |  |
| **Positive control (65 °C treatments)** | **238 (215, 239)** | **100** |  |  |  |
| **2% SDS–1% AcOH, 30 min, 65 °C** | **> 500** | **37** | **3.4 (2.8, 4.3)** | **3.5 (3.1, 4.1)** | **7.4 (6.3, > 8.5)** |
| **2% SDS–1% AcOH, 2 h, 65 °C** | **456 (426, >500)** | **50** | **3.3 (2.8, 3.9)** | **4.5 (3.8, 5.7)** | **> 8.5 (7.4, > 8.5)** |
| **2% SDS–1% AcOH, 18 h, 65 °C** | **> 500** | **31** | **3.5 (2.9, 4.4)** | **4.6 (3.8, 5.8)** | **> 8.5 (7.4, > 8.5)** |
| **Positive control (121 °C treatments)** | **257 (235, 278)** | **100** |  |  |  |
| **Untreated, 15 min, 121 °C** | **358 (307, 439)** | **100** | **2.0 (1.5, 2.5)** | **1.8 (1.3, 2.5)** | **7.1 (6.1, > 8.4)** |
| **Untreated, 30 min, 121 °C** | **427 (354, >500)** | **82** | **2.4 (1.9, 3.1)** | **> 5.7 (3.1, > 5.7)** | **> 8.3 (6.9, > 8.4)** |
| **Untreated, 2 h, 121 °C** | **389 (291, 461)** | **100** | **2.1 (1.7, 2.7)** | **> 5.7 (3.2, > 5.7)** | **> 8.3 (6.9, > 8.4)** |
| **2% SDS–1% AcOH, 15 min, 121 °C** | **> 500** | **0** | **> 3.3 (3.0, > 3.3)** | **> 5.7 (3.2, > 5.7)** | **> 8.4 (7.6, > 8.4)** |
| **2% SDS–1% AcOH, 30 min, 121 °C** | **> 500** | **0** | **> 3.3 (2.8, > 3.3)** | **3.7 (2.7, 5.5)** | **> 8.4 (7.6, > 8.4)** |
| **2% SDS–1% AcOH, 2 h, 121 °C** | **> 500** | **0** | **> 3.3 (2.9, > 3.3)** | **> 5.7 (3.2, > 5.7)** | **> 8.4 (7.7, > 8.4)** |

*^a^ Median incubation period (IP) in days, 95% confidence intervals (ci), and percentage of mice succumbing to prion disease (10 mice per treatment) were calculated by using Kaplan-Meier analysis.*

*^b^ Incubation period data previously reported [1].*

1. Peretz D, Supattapone S, Giles K, Vergara J, Freyman Y, et al. (2006) Inactivation of prions by acidic sodium dodecyl sulfate. J Virol 80: 322-331.
