# Supplementary material for: Resistance of Bovine Spongiform Encephalopathy (BSE) Prions to Inactivation
Source: PLoS Pathog. 2008 Nov 14;4(11):e1000206. doi: 10.1371/journal.ppat.1000206 (PMC2576443; doi:10.1371/journal.ppat.1000206)
Supplement: Table S2 — Inactivation of cattle BSE prions bound to stainless steel wires. (0.04 MB DOC) [file ppat.1000206.s004.doc]

Suppl. Table 2. Inactivation of cattle BSE prions bound to stainless steel wires^a^

| *Treatment* | *IP (95% ci)* | *Sick (%)* |
| --- | --- | --- |
| *Positive control (2% SDS/65 °C treatments)* | *317 (294, 324)* | *100* |
| *2% SDS–1% AcOH, 30 min, 65 °C* | *418 (327, 495)* | *90* |
| *2% SDS–1% AcOH, 2 h, 65 °C* | *> 500* | *30* |
| *2% SDS–1% AcOH, 18 h, 65 °C* | *> 500* | *10* |
| *Positive control (2% SDS/121 °C treatments)* | *308 (288, 319)* | *100* |
| *Untreated, 15 min, 121 °C* | *361 (319, 364)* | *100* |
| *Untreated, 30 min, 121 °C* | *377 (326, 476)* | *100* |
| *Untreated, 2 h, 121 °C* | *377 (319, 403)* | *100* |
| *2% SDS–1% AcOH, 15 min, 121 °C* | *> 500* | *0* |
| *2% SDS–1% AcOH, 30 min, 121 °C* | *> 500* | *0* |
| *2% SDS–1% AcOH, 2 h, 121 °C* | *> 500* | *0* |

^a^ Median incubation period (IP) in days, 95% confidence intervals (ci), and percentage of animals succumbing to prion disease (10 mice per treatment) were calculated by using Kaplan-Meier analysis.
